# Implementation of evidence into practice for cancer-related fatigue management of hospitalized adult patients using the PARIHS framework

**DOI:** 10.1371/journal.pone.0187257

**Published:** 2017-10-31

**Authors:** Li Tian, Yiqun Yang, Wenjie Sui, Yan Hu, Huiling Li, Fen Wang, Keyan Qian, Juan Ji, Min Tao

**Affiliations:** 1 The First Affiliated Hospital of Soochow University, Suzhou, China; 2 School of Nursing, Fudan University, Shanghai, China; 3 School of Nursing, Soochow University, Suzhou, China; University of Texas MD Anderson Cancer Center, UNITED STATES

## Abstract

This study aimed to explore an evidence-based nursing practice model of CRF management in hospitalized adult patients using the PARIHS evidence-implementation framework as the theoretical structure to provide guidance for similar nursing practices. The implementation of guideline evidence into clinical practice was conducted on the oncology and radiotherapy wards of a university-affiliated hospital. The process of integrating the guideline into the symptom management system of cancer patients was described. The impact of the evidence implementation was evaluated from three aspects: organizational innovations and outcome measures associated with nurses and with patients pre- and post-evidence implementation. During the implementation of evidence into practice on the wards, a nursing process, health education, a quality control sheet and CRF training courses were established. Through this implementation, compliance with evidence related to CRF increased significantly on the two wards, with that of ward B being higher than that of ward A. Regarding nursing outcomes, nursing knowledge, attitude and behavior scores with respect to CRF nursing care increased substantially after its application on the two wards, and the ward B nurses’ scoring was higher than that of the ward A nurses. Qualitative analysis concerning the nurses suggested that leadership, patient concern about CRF management, and the need for professional development were the main motivators of the application, whereas the shortage and mobility of nursing human resources and insufficient communication between doctors and nurses were the main barriers. Additionally, most nurses felt more professional and confident about their work. Regarding patient outcomes, patient knowledge, attitude and behavior scores regarding CRF self-management increased significantly. Patients’ post-implementation CRF was alleviated compared with the pre-implementation treatment cycle. The PARIHS framework may provide instructive guidance for the incorporation of evidence into practice, and the process-oriented framework might provide greater operational utility of the application.

## Introduction

Cancer-related fatigue (CRF) has been reportedly experienced by 80%-90% of cancer patients undergoing chemotherapy or/and radiotherapy [1] and is considered more distressing to patients than either pain or nausea and vomiting [2]. Despite its high prevalence and adverse effects on the quality of life of patients and their families, CRF is rarely diagnosed and treated; moreover, it has not attracted sufficient attention from the clinical professions, cancer patients and their families. Because there are no medical regulations regarding CRF in mainland China, this condition has not been routinely screened for, assessed, and treated in the symptom management of cancer patients to date. A growing and persuasive body of evidence on CRF (including guidelines, systematic reviews, and meta-analyses) is accumulating. Considering the differences in internal and external cultures and clinical situations [3–6] along with abundant indigenous evidence resources, we developed the “Clinical Practice Guideline: Nursing Care of Cancer-Related Fatigue in Adults with Cancers” under the guidance of SIGN 50 (a guideline developer’s handbook) issued by the Scottish Intercollegiate Guidelines Network (SIGN) [7]. The current need regards implementing this evidence into CRF management. Implementation of evidence into practice is an iterative and gradual process that includes actively and systematically integrating information into place, defining the barriers to the innovation, resolving these barriers by effective communication, and improving the effects of the innovation using management or educational strategies [8]. However, prior to launching the innovation, factors influencing the innovation should be identified; moreover, fundamental specialized knowledge and skills are required to support clinical decisions, guaranteeing the implementation of the innovation in a specific context [3,9]. The PARIHS (Promoting Action on Research Implementation in Health Services) framework, which includes three key elements (i.e., evidence level and characteristics, context, and facilitation), was originally conceived by Kitson et al. [10] to help interconnected professionals understand the complexities involved in the successful implementation (SI) of evidence into practice. Additionally, it outlines the factors that require attention before, during, and after the implementation process, which may guarantee the successful application of the evidence [11]. Therefore, we adopted the PARIHS framework to guide the implementation process.

Our goal was to explore an evidence-based nursing practice model of CRF management in hospitalized adult patients during the clinical application of the “Clinical Practice Guideline: Nursing Care of Cancer-Related Fatigue in Adults with Cancer” (hereinafter referred to as the CRF nursing guideline) using the PARIHS framework of evidence implementation as the theoretical construct to provide guidance for similar nursing practices.

## Methods

This study acquired the ethics approval from the medical ethics committee of the First Affiliated Hospital of Soochow University on 25th March 2014, and the ID is 2014–147. All participants signed the informed consent.

**Fig 1:**
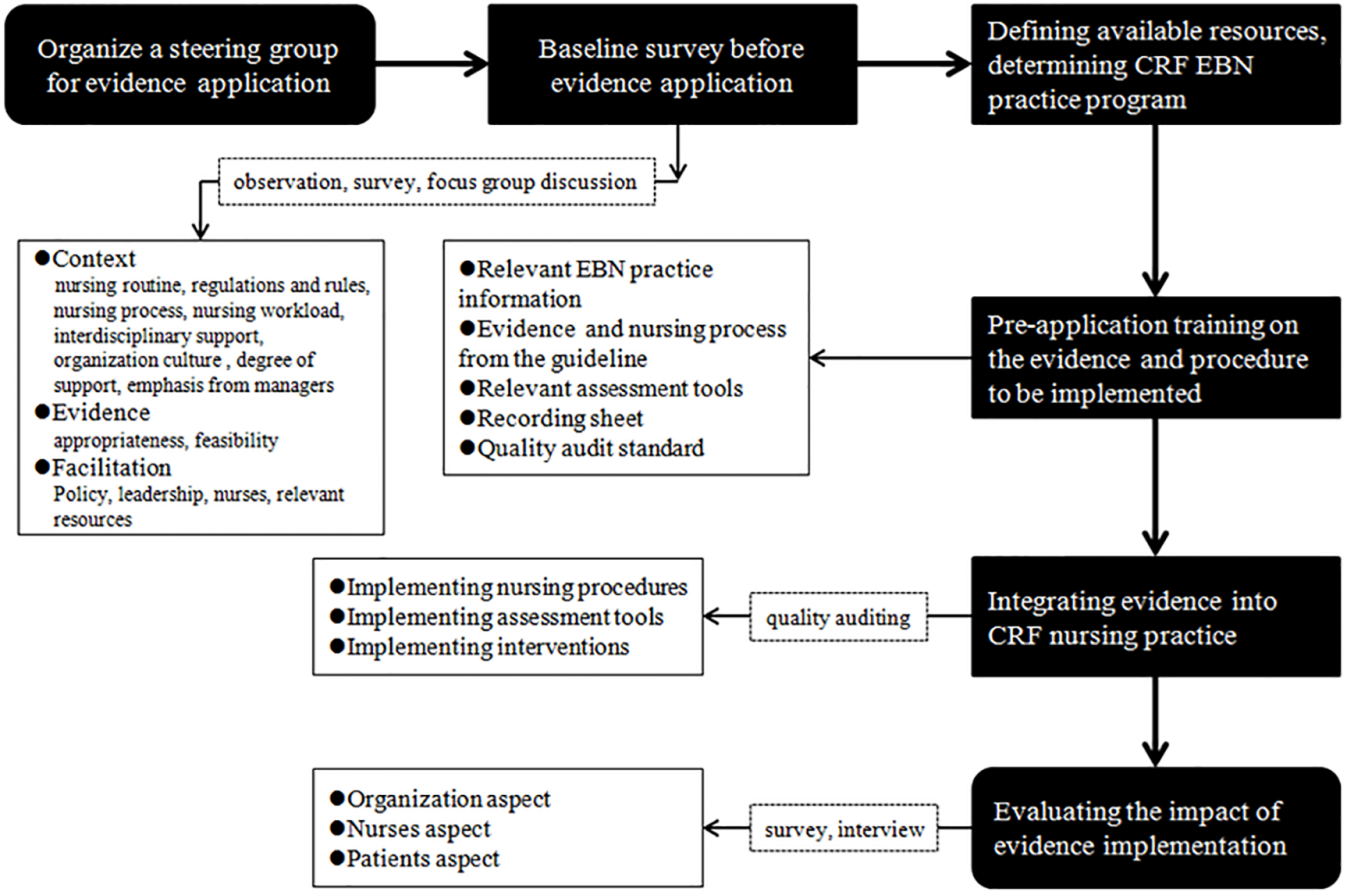
The structure of evidence application process.

This evidence application project in the departments of medical oncology and radiotherapy of a university-affiliated adult hospital in Suzhou adopted a pre- and post-test design. Although most of the patients from the two units experienced clinically significant CRF, CRF had not been consistently screened for, assessed and treated as a dependent symptom [12]. Baseline surveys and related preparatory activities were conducted from May to July 2015, and clinical application was performed from July to September 2015. During this process, we organized several seminars on evidence-based nursing practice concerning CRF management (i.e., discussions among steering group members, and training sessions of nursing professionals), gradually applied the related evidence into the routine care of cancer patients’ symptom management, described the process of integrating the evidence into nursing practice, and evaluated the changes in outcome indicators of the system, nurses and patients (Fig 1).

### Organize a steering group for evidence application

The steering group was composed of six directors from the nursing department, department of medical oncology, department of radiotherapy, department of Traditional Chinese Medicine, and department of psychiatry and two researchers from the Evidence-Based Nursing (EBN) Collaborating Center of the Joanna Briggs Institute, Fudan University.

### Baseline survey

#### Context

The characteristics of the context were assessed using the observational method, including nursing routines or procedures, current regulations and rules related to the proposed evidence to be implemented, nursing workload, interdisciplinary support, organization culture of the research site, degree of support, and emphasis from managers.

#### Evidence

The evidence in the CRF nursing guideline derived from foreign professional institutions' guidelines [13], systematic reviews [14–17], meta-analyses [18–26], randomized controlled trials [27–32] and descriptive research [33]. Based on a comprehensive analysis of the strengths and weaknesses of current standards concerning the quality of evidence rating and the strength of recommendations “The Oxford Levels of Evidence” [34] and “The Joanna Briggs Institute Grades of Recommendation” [35] were chosen as the standards for rating all the included evidence. All the ratings were assessed by all the panel members who developed the CRF nursing guideline.

#### Facilitation

Evidence application facilitations and barriers were evaluated and defined using focus group discussions. These factors were organized, analyzed and discussed to develop countermeasures.

### Defining available resources, determining CRF EBN practice program

A steering group symposium focused on the baseline survey assessment results (including **C**ontext, **E**vidence and procedure, **F**acilitation during evidence application) was held to define the available resources (support from professional teams, health information and platform resources), formulate proposed changes, and determine a CRF evidence-based nursing practice program.

### Pre-application training on the evidence and procedure to be implemented

The pre-application training included the concept of evidence-based nursing; the necessity for CRF EBN practice implementation; the content, source, quality, and strength of the evidence recommendations; the related practice procedures to be implemented; the assessment tools to be used; and the quality review standards of CRF nursing practice. Relevant print materials were distributed to the nurses who participated in the evidence application. At any time during and after the training, the nurses were permitted to ask questions and make suggestions. The steering group members organized and analyzed these suggestions and opinions to determine whether modifications to the nursing procedure and quality review sheet were needed.

### Integrating evidence into CRF nursing practice, periodic feedback and summary

The integrated evidence included the CRF screening and assessment procedures and tools, CRF interventions, and quality review sheet. The steering group members conducted rounds on the units periodically, addressed challenges, and collected feedback and suggestions posed by the professionals at the research site. The feedback and suggestions were collated, and common problems were analyzed to determine whether a specific training was required. We identified the interventions that could not be implemented smoothly and analyzed the reasons by re-evaluating the feasibility and appropriateness of the evidence and the characteristics of the context. Then, we considered whether the evidence could be implemented into CRF nursing practice.

### Data collection

We evaluated the impact of evidence implementation from three aspects: organizational innovations, and outcome measures related to nurses and patients pre- and post-implementation of the interventions.

#### Organization

We assessed the changes in CRF nursing practice, including those concerning nursing procedures, nursing education, related nursing training, and quality control, using the methods of conversation and observation. Additionally, we identified compliance with the evidence-based practice (EBP) using a “nursing quality checklist of inpatient CRF management” to evaluate the changes in CRF management on the pilot units.

#### Nurses

At the end of the clinical application, maximum difference sampling was used to select nurses to assess the nurses’ experiences and insights regarding this EBP project using semi-structured interviews. The included nurses were those who (1) had worked in the pilot unit for at least one year; (2) were fully engaged in this EBP project and (3) were willing to participate in the interview. COREQ criteria were used for the reporting of this qualitative research [36]. After signing written informed consent, a 30–60 min in-depth individual face-to-face interview was conducted by T.L., including audio-taping and transcribing in full and de-identification. All transcribed data were collated and returned to the interviewee for verification. All interviews were conducted during working hours at the hospital using an interview guide that covered a range of questions including the following: (1) what was your feeling when you knew your ward would carry out this innovation project? (2) what were your suggestions when you made the comparison between the recommended evidence and the current CRF management interventions, and what did you think then? (3) when management made the changes to the nursing process and carried out the pre-application training for the nurses, what was your opinion, and did you accept the altered processes? (4) what problems you have encountered when the new process was carried out in your ward? (5) the pilot application phase is coming to an end, what do you think the effect of the evidence implementation will be? What were your results? (6) what factors (facilitating or impeding) affected this EBP project? The interview guide was developed based on the literature review and a shared discussion among the steering group. The interviews continued until the point of data saturation, i.e., when no new themes pertinent to the study’s core foci were emerging [37].

The pre- and post-implementation changes in nurses’ knowledge, attitudes and behaviors regarding CRF (NKAB-F) were surveyed using the 35-item scale developed based on the clinical guideline of CRF nursing care. This 3-point scale was reviewed by experts in evidence-based practice and oncology nursing, and its content validity index (CVI) was 0.96, indicating that this scale had good content validity [38].

#### Patients

Patients’ outcome measures included two aspects: CRF self-management ability (including knowledge, attitudes and behaviors) and the changes in CRF scaling. The former was assessed using the 3-point (ranging from 1 = to no extent to 3 = to great extent) and 16-item “CRF self-management scale” (which was also reviewed by experts in oncology nursing; its CVI was 0.98). Self-management ability was also assessed using the 6-item “self-efficacy questionnaire for CRF management (SQFM)”, which was adapted from the “Self-efficacy Questionnaire for Cancer Patients, SQCP” [39]; its CVI was 0.95 with a coefficient of internal consistency of 0.96. The inclusion criteria were as follows: (1) adult cancer patients (aged ≥18 years) with a pathology- or cytology-based diagnosis and aware of their diagnosis; (2) no cognitive impairment; and (3) willingness to participate in the survey. The changes in CRF scaling were collected using the data from the nursing records and patients’ CRF diaries.

### Data analysis

Descriptive statistics were used to analyze the quantitative data related to the outcomes of the institution, nurses and patients. Provided the evidence was well recognized, acknowledged and implemented by all key players, the innovation in the health service delivery and organization could be conducted smoothly [40]. Therefore, in analyzing the scale of knowledge, attitudes and behaviors regarding CRF of the nurses and patients, we calculated the percentage of scores in the 3 range (to great extent) for each item and compared the difference between the two pilot units pre- and post-implementation using the chi-square test and Fisher’s exact test. The difference of nurses’ compliance of the EBP between the two pilot units was compared using Fisher’s exact test. Using *t* tests, the CRF scales and patients’ CRF self-management scales were compared between the two pilot units. Qualitative content analysis was conducted on verbatim transcripts of the semi-structured interviews. First, researchers read the transcripts carefully, extracted and marked the meaningful statements according to the interview outline, coded and classified these statements, and refined the themes using generic analysis methods [41]. Independent coding (T.L. and H.Y.) was cross-checked to develop themes from the data (L.HL. and S. WJ.), moving toward an overall interpretation of the data. This cycle was repeated until no additional new themes were found. Inter-rater reliability was ensured by including two clinical nursing specialist (L.HL. and S. WJ.) in addition to two researchers (T.L. and H.Y.).

## Results

### Characteristics of the pilot units

Unit A had 18 nurses (Table 1), 42 beds and 490 monthly turnover cases, and the average length of stay was 3.3 days. This unit mainly received gastro-enteric cancer patients for adjuvant chemotherapy. Unit B had 21 nurses (Table 1), 40 beds and 180 monthly turnover cases, and the average length of stay was 8.5 days. This unit mainly received nasopharynx, lung and esophagus cancer patients for adjuvant radiotherapy.

**Table 1:** Characteristics of nurse participants.

Characteristic	Number(%)	
	Ward A(n = 18)	Ward B(n = 21)
Age (years, mean±SD)	31.00±6.97	31.33±7.80
Gender		
Female	18(100)	21(100)
Male	0	0
Educational level		
Post-graduate	0	1(4.76)
University	6(33.33)	13
Junior college	10(55.56)	6
Technical secondary school	2(11.11)	1(4.76)
Professional titles		
Co-chief superintendent nurse	2(11.11)	0
Charge nurse	2(11.11)	7(33.33)
Senior nurse	7(38.89)	11(52.38)
Nurse	7(38.89)	3(14.29)
Years working in the current ward		
<5 years	9(50.00)	12(57.14)
5~10 years	4(22.22)	4(19.05)
>10 years	5(27.78)	5(23.81)

### Organizational changes

**Fig 2:**
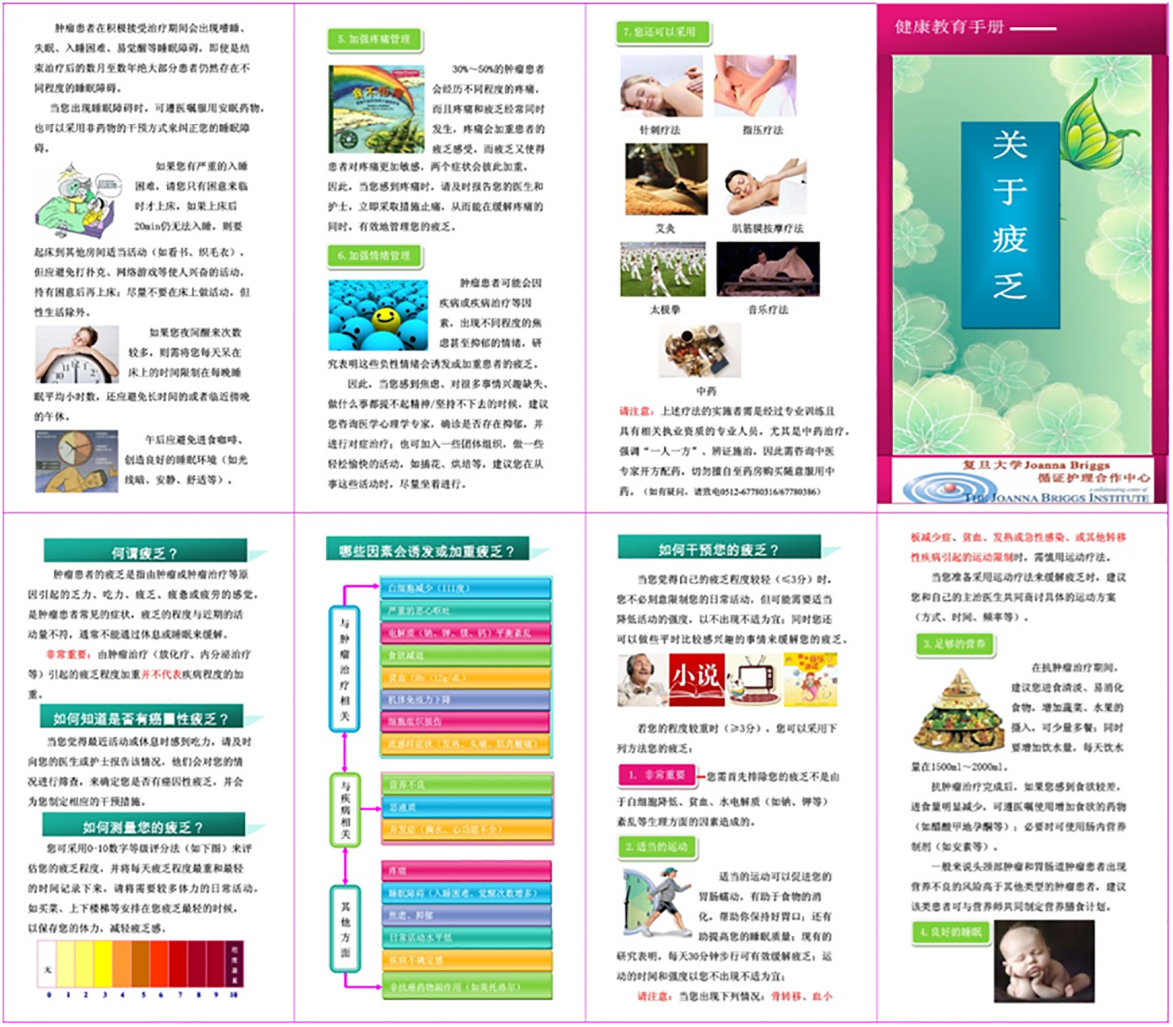
CRF health education handbook.

**Fig 3:**
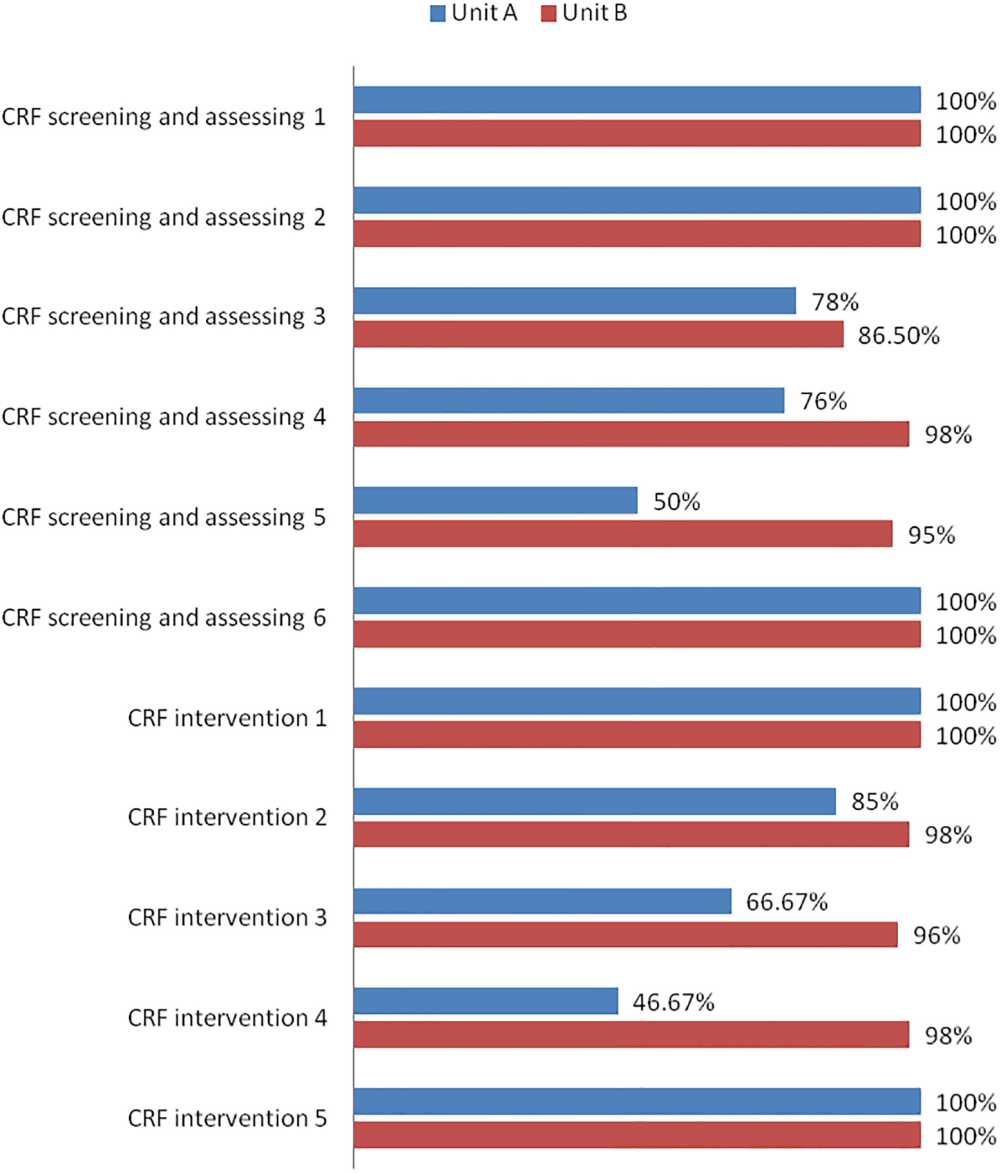
Compliance of correlated evidence in units A and B.

Before the EBP, CRF had not been addressed by medical professionals and patients, and no nursing procedures or record sheets existed regarding CRF. Through the EBP implementation, a CRF nursing procedure (including screening and assessment) and an intervention procedure were developed. A nursing record chart, CRF health education handbook (Fig 2), and CRF quality control checklist were formulated. Initial training courses on CRF nursing care were established, including elementary training on evidence-based nursing practice and specific training on CRF nursing care. The project implementation of the two pilot units was evaluated by testing for compliance with the EBP, which indicated that unit B was superior to unit A (Fig 3).

### Nurse outcomes

Twelve nurses with a mean age of 32.25 (24–45) years participated in the semi-structured interviews: 3 were nurse leaders, 6 had bachelor’s degrees, 1 had a post-graduate degree, 5 had worked in the current unit for less than 3 years, and 4 had worked on the unit for longer than 10 years. The nurses’ experiences and insights were refined and organized into the following five themes:

- During the inception phase, the nurses had different attitudes towards the EBP project, including approval and rejection
  “……*in the past, most of our nursing care plan was still empirical……while this EBP project contributes to improve the reputation of our specialized nursing, we highly appreciate this project*.”
  “*I feel there will be another project, which will increase the workload, in the beginning, I think it’s troublesome, because there are too many forms to fill out*.”
- During the application process, the primary problems encountered by the nurses were the following: the burden of completing the forms, the patients’ poor comprehension, and poor compliance with the interventions
  “*Many patients reported that the burden of completing the form was too great; there were many questions to answer. It would be better if they only had to fill in the form on the CRF assessment*.”
  “*The most significant problem is hard to communicate because most of our patients have lower education levels and their ability to comprehend the items on these scales is very poor……therefore it (filling out the form) takes me too much time*”
  “*Some patients showed poor treatment compliance, they had a good response to what you said when you gave the nursing education; however, they seldom follow what you have told them*.”
- During this application process, the effective facilitations included strong leadership, patients attaching great importance to CRF management, and nurses’ need for professional growth
  “*The head nurse often makes the rounds of CRF nursing care, then staff nurses will make comparisons, if some of them do better than others, other nurses will then try their best*.”
  “*By embedding the CRF nursing care in the quality control system of our hospital, in the oncology nursing standards, then we nurses will feel these interventions on CRF management should be carried out*.”
  “*If the patients have a need for CRF management, or if they pay enough attention to the CRF management, I am sure I will apply these nursing interventions to them*.
  “……*patients increase their reliance on me……which will improve my professional confidence, which pushes me to give CRF nursing education to more patients*.”
- During this application process, the main barriers included the relative shortage and great mobility of nurse human resources and inadequate communication between doctors and nurses
  “*the main barriers are shortage of time and heavy workload……28 turnover cases daily……there are 4 nurses on sick leave recently, whether me or other nurses, if we have time, we would like to do this (EBP of CRF management)*”.
  “*Even if the number of nurses met standards, the mobility of nurses is great, especially for those experienced nurses, so there are many fresh nurses in our unit*……”
  “*Some doctors don’t grasp the core knowledge of CRF management…… for example, according to the guideline, exercise should be recommended to CRF patients to alleviate the degree of CRF, while some doctors still advise the patients to rest, and then all our efforts were in vain because the patients relied on the doctors more than the nurses*.”
- Throughout this EBP project, the nurses’ main achievements were heightened professionalism and greater confidence
  “*This EBP project is beneficial for our work. If patients’ CRF scores are high, we will pay more attention to them. Through the intervention implementation process, we now know how to communicate with the patients about their CRF, and how to give CRF nursing education; then, with patients showing increased reliance on us, I’ve become more confident in my performance, and I believe that this is helpful to improve specialized nursing*.”

Thirty nurses from the two units participated in the pre-implementation survey, whereas three nurses left the units due to occupation mobility; therefore, only 24 nurses completed the post-implementation survey. The NKAB-F scale examined the changes in nurses’ knowledge, attitudes and behaviors regarding CRF management.

Before the EBP project, the NKAB-F scores were not significantly different between the two pilot units. After implementation of the EBP project, the knowledge, attitude and behavior scores were all higher than at baseline, and the unit B scores were higher than those of unit A.

### Patient outcomes

Fifty patients from unit A and 55 patients from unit B completed the pre-implementation survey, and 44 patients from unit A and 55 patients from unit B completed the post-implementation survey. No differences were detected between the baseline and final scores of the SQFM scale for both unit A [31.38(5.88) vs. 29.34(4.03); t = 1.93, p = 0.06] and unit B [23.67(6.25) vs. 21.09(8.23); t = 1.85, p = 0.07]. Regarding CRF self-management ability, significant differences were found for patient knowledge, attitude and behavior scores between the baseline and final evaluation in both unit A and unit B (Table 2).

**Table 2:** Patients’ scores of CRF self-management scale pre- and post- implementation.

Unit	Pre-implementation (n = 105) mean±SD	Post-implementation (n = 99) mean±SD	t	p
Scaling of knowledge				
A	34.74±7.07	38.70±6.21	-2.87	<0.01
B	35.11±6.01	41.05±6.09	-5.15	<0.01
Scaling of attention				
A	29.72±4.05	35.41±5.51	-5.64	<0.01
B	33.51±6.17	37.51±5.70	-3.53	<0.01
Scaling of behavior				
A	31.00±3.63	36.36±4.91	-5.96	<0.01
B	33.24±4.57	37.42±5.56	-4.31	<0.01

During the implementation, a total of 30 patients completed the CRF diary. Using a paired *t* test, the most severe CRF scores (using a 0–10 fatigue scale) during the treatment cycle that included the EBP project were lower than those of the prior treatment cycle [5.59(2.09) vs. 6.50(1.90); t = 2.22, p = 0.04]. Before implementation, most cancer patients rested to alleviate their CRF, and after the EBP project, patients who had received CRF nursing education (which was developed based on the CRF nursing guideline evidence) had adopted more effective interventions for their CRF. According to their diaries, we found that the most frequent interventions to relieve CRF adopted by the patients during the treatment interval included exercise (n = 14, all were walking), Traditional Chinese Medicine (n = 11), taking drugs to improve appetite (n = 6), and massage (n = 6). Through these interventions, their CRF levels were effectively alleviated.

## Discussion

From this EBP project, we confirmed the feasibility and effect of the EBN scheme based on the CRF clinical guideline and identified the following critical factors that influenced the SI of evidence into practice through a comprehensive examination and analysis of the entire application process.

First, formulating simple and effective strategies and interventions of the innovation can guarantee the SI of evidence into practice. Because “high” evidence is the only basic element in the inception phase of the implementation in clinical practice [11,42], the complexity of these strategies and interventions directly influence the SI of evidence into practice; therefore, they should be as simple and effective as possible to facilitate the target population’s ability to learn, accept and implement them. For our EBP, we developed a CRF nursing education handbook with many graphics based on the core tenets of the CRF guideline. We sent this handbook to the patients and placed one copy on each unit to facilitate patient and caregiver learning about CRF management. Additionally, during the implementation, not only should the implementation of the evidence and its strategies be considered but also its appropriateness (i.e., to patient heterogeneity and special contexts). Therefore, the “best practice” may not be suitable for everyone.

Second, “high” context contributes to the SI of evidence into practice. Regarding “contexts conducive to change” [11,42], we summarized four key sub-elements: sufficient nurse human resources, professionals receptive to change, the EBP fit with the strategic intent and goals of the organization, and strong leadership. In China, nurse human resources are allocated according to the “bed to nurse ratio” (BNR), and in this study, the BNR of unit A (0.45) and unit B (0.525) met the national standard, whereas the turnover cases (per month) in unit A (approaching 500) was three times that of unit B. Although nurses in unit A reported that they believed that these interventions were very important and useful, their compliance with the implementation was clearly lower than that of unit B. Therefore, sufficient nurse human capital effectively ensured the SI. Additionally, the number of novice nurses was similar in the two units, whereas the nurses’ education level in unit B was higher than that in unit A, which resulted in more receptiveness to change in unit B than in unit A during the implementation inception stage. Communication theory applicable to innovation in the healthcare field indicated that communication became more effective when the information disseminators and information recipients shared the same values and faith [38]. Our EBP project fit with the reputational construct needs of specialized nursing in this setting; therefore, we received substantial support from the nursing management, which ensured the SI to some extent. Regarding “strong leadership”, we found that the leadership was not limited to the nursing management:, it also included the “opinion leader” in the study setting, whose opinions were often repeated and accepted by other nurses and whose behaviors were often imitated by other nurses. Particularly during the inception stage of the implementation when most of the staff nurses rejected the innovation, we identified the “opinion leader” in the study setting [43], who tried to persuade and train the other nurses and could “act as a role model” for nursing practice, which helped ensure the SI. Therefore, the role of an “opinion leader” appeared crucial in the SI of evidence into practice.

Third, the conversion of “external causes” into “internal causes” was fundamental to ensure the sustainability of the SI of evidence into practice [44]. The difficult part of evidence application rests in how to achieve sustainable implementation of the evidence. Currently, many studies have focused on strategies that potentially facilitate the implementation process and related resources, for example, by including EBP experts and advocates, developing multi-disciplinary teams, providing sufficient time, resources and associated support [45,46]. With our EBP, because the hospital administrators were highly focused on the reputational construct of specialized nursing, the nursing unit leaders were required to execute this effort; therefore, the facilitators of the implementation were not only the university investigators (external causes) but also the nursing leaders in the nursing department and units (internal causes), which enabled the sustainability and integration of these innovations into the routine nursing care on the pilot unit even after the completion of our EBP project. Thereby, the staff nurses developed professional confidence and competencies through the implementation, which also ensured the sustainable implementation of the evidence.

Finally, we examined the EBP practice based on the PARIHS framework. We divided the entire evidence application process into four stages: the preparation stage, inception stage, transitional stage and sustainable stage. During the preparation stage, the primary facilitators were the investigators, and the importance of the clinical problems to be resolved and the necessity of implementing the clinical innovations were emphasized. The leading factors were the evidence quality level and characteristics (feasibility, appropriateness, meaningfulness and effectiveness) (E) [47]. During the inception stage, the primary facilitators were the administrators and investigators, and the emphasis was to create a “high” context, including having sufficient human resources and strong leadership, identifying the “opinion leader” and persuading this leader to accept the innovation. The leading factor was the “high” context (C) rather than evidence. During the transitional stage, the primary facilitators were the investigators and administrators and the leading factor was evidence because at this stage, clinical professionals make evidence-informed decisions, which are related to the feasibility, appropriateness, and significance of the evidence. This phase serves as a bridge between the preceding and following stages because this stage may revert back to the inception stage or proceed to the sustainable stage. For example, the relative shortage of nurse human resources may hinder the implementation, whereas professional confidence may facilitate the implementation. During the sustainability stage, the primary facilitators were the administrators, and the leading factor was the facilitations (F), including the guarantee of the policy and regulations, initiation of the motivating power inside the organization, and supervision and feedback outside the organization.

## Conclusions

The implementation structure incorporating CRF guidelines into clinical practice using the PARIHS framework demonstrated some feasibility, appropriateness and effectiveness and might be used as a reference for the clinical utilization of other similar guidelines. The PARIHS framework may provide instructive guidance for incorporating evidence into practice, and the process-oriented framework might provide greater operational utility of the application.

### Limitations

Due to the limitations of time and funds, the EBP was only conducted on two units, and the clinical practice implementation of evidence only continued for two months. The observation of patient outcomes only continued for one treatment cycle (one month). Additionally, this EBP project encountered a relative shortage of human resources in the setting, which might weaken other influential factors. In future research, a multi-center evidence application, a longer implementation process and outcome observations should be performed to examine the long-term application effects of the EBP project.

## Supporting information

S1 Table CRF Nursing quality checklist -Chinese version. (DOCX)

S2 Table CRF Nursing quality checklist -English version. (DOCX)

S3 Table NKAB-F-Chinese version. (DOCX)

S4 Table NKAB-F-English version. (DOCX)

S5 Table Patients’ CRF self-management scale-Chinese version. (DOCX)

S6 Table Patients’ CRF self-management scale-English version. (DOCX)

S7 Table SQFM-Chinese version. (DOCX)

S8 Table SQFM-English version. (DOCX)
