# Supplementary material for: Implementation of evidence into practice for cancer-related fatigue management of hospitalized adult patients using the PARIHS framework
Source: PLoS One. 2017 Oct 31;12(10):e0187257. doi: 10.1371/journal.pone.0187257 (PMC5663504; doi:10.1371/journal.pone.0187257)
Supplement: S1 Table — (DOCX) [file pone.0187257.s001.docx]

### 住院患者癌因性疲乏症状护理质量审查表

| 病区： | 审查时间：_ 年___月___ 日 | | 审查人： |
| --- | --- | --- | --- |
| 住院号： | 诊断： | | 癌因性疲乏：□有 □无 |
| 肿瘤治疗方式：□放疗 □化疗 □放化疗 | | 肿瘤治疗周期：□治疗期 □治疗间歇期 | |

| 项 目 | 内 容 | 评价形式 | 评价结果 | 备 注 |
| --- | --- | --- | --- | --- |
| 癌  因  性  疲  乏  的  筛  查  和  评  估 | 1. 护士是否按照癌因性疲乏评  估流程对患者进行评估 | 现场查看 | □是 □否 |  |
|  | 2. 护士是否对初次来病房接受  治疗的患者进行癌因性疲乏的诊  断筛查 | 查看病历 | □是 □否 | 选用的诊断筛查工具为：  □ICD-10 CRF诊断工具  □其它:______________ |
|  | 3. 患者能否进行癌因性疲乏的  自我评估 | 现场询问 | □能 □否 |  |
|  | 4. 患者住院接受治疗期间，护  士是否每天对患者的疲乏程度进  行评估 | 现场查看  查阅病历 | □是 □否 | 选用的工具为：  □0-10数字等级评分  □其它:______________ |
|  | 5. 对中度以上疲乏患者护士是  否进行与疲乏相关的危险因素评  估 | 查阅病历 | □是 □否 |  |
|  | 6. 评估结果是否完整，包括患  者自我报告的评估内容 | 查阅病历 | □是 □否 |  |
| 癌  因  性  疲  乏  症  状  管  理 | 1. 患者是否知晓癌因性疲乏的一般性干预措施 | 现场询问  查阅病历 | □是 □否 | 已采取的一般性干预措施（可多选）：  □健康教育  □运动疗法  □物理疗法（针刺、针压、艾灸、按摩）  □音乐疗法 |
|  | 2. 患者是否知晓癌因性疲乏的  对症处理措施 | 现场询问  查阅病历 | □是 □否 | 已采取的对症处理措施（可多选）：  □升白治疗  □止吐治疗  □纠正水电解质紊乱  □使用食欲刺激剂（如醋酸甲地孕酮或安宫黄体酮）  □止痛  □处理并发症（如抽胸水）  □纠正贫血  □抗抑郁治疗  □纠正睡眠障碍  □心理护理（认知行为疗法、团体支持-表达治疗、正念减压法） |
|  | 3. 患者是否知晓癌因性疲乏的  支持治疗措施 | 现场询问  查阅病历 | □是 □否 | 已采取的支持治疗措施（可多选）：  □营养支持治疗  □中药治疗 |
|  | 4. 护士是否对癌因性疲乏的干  预效果及时进行评价 | 查阅病历 | □是 □否 | 评价工具：  □0-10数字等级评分  □ BFI  □ FACT-F  □ PFS-R  □其它:______________ |
|  | 5.护士是否对拟出院的癌因性疲  乏患者进行健康教育 | 现场询问  查阅病历 | □是 □否 |  |

注：请在符合的选项前的方框内打“√”。
