# Supplementary material for: Implementation of evidence into practice for cancer-related fatigue management of hospitalized adult patients using the PARIHS framework
Source: PLoS One. 2017 Oct 31;12(10):e0187257. doi: 10.1371/journal.pone.0187257 (PMC5663504; doi:10.1371/journal.pone.0187257)
Supplement: S2 Table — (DOCX) [file pone.0187257.s002.docx]

### Nursing quality checklist of inpatient CRF management

| Ward ： | Auditing date：_____/____/____ | | Auditor： |
| --- | --- | --- | --- |
| Admission No.： | Diagnosis： | | CRF：□YES □NO |
| Treatment：□radiotherapy □chemotherapy □radiotherapy and chemotherapy | | Treatment status：□on-going □off-treatment | |

| Item | Contents | Form of evaluation | Evaluation result | Notes |
| --- | --- | --- | --- | --- |
| Screening  And  Evaluation  Of CRF | 1. whether the nurse assesses the patient’s CRF according to the assessment procedure | On-site observation | □YES □NO |  |
|  | 2. whether the nurse screens the patient's CRF at their initial visit | View medical records | □YES □NO | Screening tools:  □ICD-10 -CRF  □others:______________ |
|  | 3. whether the patient can self-assess their own CRF | On-site inquiry | □YES □NO |  |
|  | 4. whether the nurse assesses the patient’s CRF daily during their hospitalization | On-site observation  View medical records | □YES □NO | Measurements:  □0-10 numeric rating scale  □others:______________ |
|  | 5. whether the nurse assesses the contributing factors of CRF when the patient experiences moderate CRF or above | View medical records | □YES □NO |  |
|  | 6. whether the assessment is complete, including the patient’s self-report | View medical records | □YES □NO |  |
| Interventions  of CRF | 1. whether the patient knows about the common interventions of CRF | On-site inquiry  View medical records | □YES □NO | Common interventions that have been taken（multiple choices possible）:  □health education  □exercise therapy  □physical therapy（acupuncture, acupressure, moxibustion, massage）  □music therapy |
|  | 2. whether the patient knows about the symptom management of CRF | On-site inquiry View medical records | □YES □NO | Symptom management interventions that have been taken（multiple choices possible）:  □increase WBC  □stop vomiting  □correct water and electrolyte disorder  □use appetite stimulants, such as megestrol acetate and medroxyprogesterone acetate  □relieve pain  □treat complications, such as chest drainage  □treat anemia  □treat depression  □correct sleep disorders  □psychological care (cognitive behavioral therapy, group supportive expressive therapy, mindfulness-based stress reduction, etc） |
|  | 3.whether the patient knows about the supportive treatment of CRF | Site inquiry View medical records | □YES □NO | supportive interventions that have been taken（multiple choices possible）:  □nutrition support therapy  □Chinese herbal therapy |
|  | 4. whether the nurse evaluates the effect of the interventions on CRF timely | View medical records | □YES □NO | Measurements:  □0-10 numeric rating scale  □BFI  □ FACT-F  □PFS-R  □others:______________ |
|  | 5. whether the nurse provides health education to patients to be discharged | On-site inquiry View medical records | □YES □NO |  |

Notes : Please tick (“√”) the appropriate option.
