# Supplementary material for: Implementation of evidence into practice for cancer-related fatigue management of hospitalized adult patients using the PARIHS framework
Source: PLoS One. 2017 Oct 31;12(10):e0187257. doi: 10.1371/journal.pone.0187257 (PMC5663504; doi:10.1371/journal.pone.0187257)
Supplement: S3 Table — (DOCX) [file pone.0187257.s003.docx]

**护理人员癌因性疲乏护理知识、态度、行为调查表**

您好！首先非常感谢您参加此项调查，以下条目主要是关于您对癌因性疲乏护理知识、态度和行为的调查，请您逐项在最符合您的分值上打“√”。

1. 您的出生年月： 年 月

2. 您来肿瘤科工作的时间是： 年 月

3. 您目前的教育背景： 学历

□研究生 □本科 □大专 □中专

4. 您目前的职称是：

□主任护师 □副主任护师 □主管护师 □护师 □护士

5. 您目前担任的职务是：

□护士长 □带教 □专职护士

| **序 号** | **条 目** |
| --- | --- |
| 筛 查 评 估 | |
| **1** | **护士应该在患者就诊时对其进行癌因性疲乏症状筛查** |
|  | □ 3=了解 □ 2=了解一些 □ 1=不了解 |
|  | □ 3=非常重要 □ 2=比较重要 □ 1=不重要 |
|  | □ 3=完全做到 □ 2=有时做到 □ 1=从未做到  未完全做到的原因：□增加工作量 □做与不做效果一样  □想到做就做 □其他:____________ |
| **2** | **癌因性疲乏可由患者进行自我评估** |
|  | □ 3=了解 □ 2=了解一些 □ 1=不了解 |
|  | □ 3=非常重要 □ 2=比较重要 □ 1=不重要 |
|  | □ 3=完全做到 □ 2=有时做到 □ 1=从未做到  未完全做到的原因：□增加工作量 □做与不做效果一样  □想到做就做 □其他:____________ |
| **3** | **护士可教会患者自行评估癌因性疲乏的方法** |
|  | □ 3=了解 □ 2=了解一些 □ 1=不了解 |
|  | □ 3=非常重要 □ 2=比较重要 □ 1=不重要 |
|  | □ 3=完全做到 □ 2=有时做到 □ 1=从未做到  未完全做到的原因：□增加工作量 □做与不做效果一样  □想到做就做 □其他:____________ |
| **4** | **应采用ICD-10的诊断标准进行癌因性疲乏的筛查** |
|  | □ 3=了解 □ 2=了解一些 □ 1=不了解 |
|  | □ 3=非常重要 □ 2=比较重要 □ 1=不重要 |
|  | □ 3=完全做到 □ 2=有时做到 □ 1=从未做到  未完全做到的原因：□增加工作量 □做与不做效果一样  □想到做就做 □其他:____________ |
| **5** | **在抗肿瘤治疗期间医护人员应每天对患者进行癌因性疲乏的筛查评估** |
|  | □ 3=了解 □ 2=了解一些 □ 1=不了解 |
|  | □ 3=非常重要 □ 2=比较重要 □ 1=不重要 |
|  | □ 3=完全做到 □ 2=有时做到 □ 1=从未做到  未完全做到的原因：□增加工作量 □做与不做效果一样  □想到做就做 □其他:____________ |
| **6** | **抗肿瘤治疗结束后医护人员应定期随访以评估患者癌因性疲乏程度** |
|  | □ 3=了解 □ 2=了解一些 □ 1=不了解 |
|  | □ 3=非常重要 □ 2=比较重要 □ 1=不重要 |
|  | □ 3=完全做到 □ 2=有时做到 □ 1=从未做到  未完全做到的原因：□增加工作量 □做与不做效果一样  □想到做就做 □其他:____________ |
| **7** | **需要反复多次评估疲乏时，可采用0-10数字等级评分尺** |
|  | □ 3=了解 □ 2=了解一些 □ 1=不了解 |
|  | □ 3=非常重要 □ 2=比较重要 □ 1=不重要 |
|  | □ 3=完全做到 □ 2=有时做到 □ 1=从未做到  未完全做到的原因：□增加工作量 □做与不做效果一样  □想到做就做 □其他:____________ |
| **8** | **在进行全面评估和干预效果评价时，可根据目标人群的特征和评价的频次选择相应的评估工具** |
|  | □ 3=了解 □ 2=了解一些 □ 1=不了解 |
|  | □ 3=非常重要 □ 2=比较重要 □ 1=不重要 |
|  | □ 3=完全做到 □ 2=有时做到 □ 1=从未做到  未完全做到的原因：□增加工作量 □做与不做效果一样  □想到做就做 □其他:____________ |
| **9** | **对中度及以上（≥4分）疲乏患者应进行影响因素的全面评估，识别导致癌因性疲乏发生或加重的因素，并及时采取相应的干预措施** |
|  | □ 3=了解 □ 2=了解一些 □ 1=不了解 |
|  | □ 3=非常重要 □ 2=比较重要 □ 1=不重要 |
|  | □ 3=完全做到 □ 2=有时做到 □ 1=从未做到  未完全做到的原因：□增加工作量 □做与不做效果一样  □想到做就做 □其他:____________ |
| **10** | **对患者报告的发生癌因性疲乏的危险因素应进行评估和确认** |
|  | □ 3=了解 □ 2=了解一些 □ 1=不了解 |
|  | □ 3=非常重要 □ 2=比较重要 □ 1=不重要 |
|  | □ 3=完全做到 □ 2=有时做到 □ 1=从未做到  未完全做到的原因：□增加工作量 □做与不做效果一样  □想到做就做 □其他:____________ |
| 干 预 | |
| 一般性干预措施 | |
| **11** | **对肿瘤患者应进行癌因性疲乏相关知识的健康教育和咨询，内容可包括疲乏的评估方法、影响因素、干预措施等** |
|  | □ 3=了解 □ 2=了解一些 □ 1=不了解 |
|  | □ 3=非常重要 □ 2=比较重要 □ 1=不重要 |
|  | □ 3=完全做到 □ 2=有时做到 □ 1=从未做到  未完全做到的原因：□增加工作量 □做与不做效果一样  □想到做就做 □其他:____________ |
| **12** | **对肿瘤患者应采用个性化的健康教育方案来进行疲乏管理** |
|  | □ 3=了解 □ 2=了解一些 □ 1=不了解 |
|  | □ 3=非常重要 □ 2=比较重要 □ 1=不重要 |
|  | □ 3=完全做到 □ 2=有时做到 □ 1=从未做到  未完全做到的原因：□增加工作量 □做与不做效果一样  □想到做就做 □其他:____________ |
| **13** | **对非血液系统肿瘤的患者应采取运动疗法进行疲乏症状干预，尤其是对乳腺癌、前列腺癌患者可进行有氧运动锻炼** |
|  | □ 3=了解 □ 2=了解一些 □ 1=不了解 |
|  | □ 3=非常重要 □ 2=比较重要 □ 1=不重要 |
|  | □ 3=完全做到 □ 2=有时做到 □ 1=从未做到  未完全做到的原因：□增加工作量 □做与不做效果一样  □想到做就做 □其他:____________ |
| **14** | **提高患者对运动的依从性是提高运动疗法干预效果的关键** |
|  | □ 3=了解 □ 2=了解一些 □ 1=不了解 |
|  | □ 3=非常重要 □ 2=比较重要 □ 1=不重要 |
|  | □ 3=完全做到 □ 2=有时做到 □ 1=从未做到  未完全做到的原因：□增加工作量 □做与不做效果一样  □想到做就做 □其他:____________ |
| **15** | **对正在接受抗肿瘤治疗的患者应提供更多的支持（如医护人员提供的专业指导、咨询等），可提高此类患者的运动依从性** |
|  | □ 3=了解 □ 2=了解一些 □ 1=不了解 |
|  | □ 3=非常重要 □ 2=比较重要 □ 1=不重要 |
|  | □ 3=完全做到 □ 2=有时做到 □ 1=从未做到  未完全做到的原因：□增加工作量 □做与不做效果一样  □想到做就做 □其他:____________ |
| **16** | **对接受运动疗法的患者应发放印有运动疗法相关内容的纸质资料和运动水平监测仪器（如计步器），可有效提高患者的运动水平** |
|  | □ 3=了解 □ 2=了解一些 □ 1=不了解 |
|  | □ 3=非常重要 □ 2=比较重要 □ 1=不重要 |
|  | □ 3=完全做到 □ 2=有时做到 □ 1=从未做到  未完全做到的原因：□增加工作量 □做与不做效果一样  □想到做就做 □其他:____________ |
| **17** | **对于下列情况的患者应慎用运动疗法：骨转移、血小板减少症、贫血、发热或急性感染、或其他转移性疾病引起的运动限制** |
|  | □ 3=了解 □ 2=了解一些 □ 1=不了解 |
|  | □ 3=非常重要 □ 2=比较重要 □ 1=不重要 |
|  | □ 3=完全做到 □ 2=有时做到 □ 1=从未做到  未完全做到的原因：□增加工作量 □做与不做效果一样  □想到做就做 □其他:____________ |
| **18** | **可采用针刺疗法和指压疗法缓解患者的癌因性疲乏** |
|  | □ 3=了解 □ 2=了解一些 □ 1=不了解 |
|  | □ 3=非常重要 □ 2=比较重要 □ 1=不重要 |
|  | □ 3=完全做到 □ 2=有时做到 □ 1=从未做到  未完全做到的原因：□增加工作量 □做与不做效果一样  □想到做就做 □其他:____________ |
| **19** | **可采用局部艾灸的方法来缓解患者的癌因性疲乏** |
|  | □ 3=了解 □ 2=了解一些 □ 1=不了解 |
|  | □ 3=非常重要 □ 2=比较重要 □ 1=不重要 |
|  | □ 3=完全做到 □ 2=有时做到 □ 1=从未做到  未完全做到的原因：□增加工作量 □做与不做效果一样  □想到做就做 □其他:____________ |
| **20** | **对康复期肿瘤患者可采用肌筋膜按摩疗法来减轻疲乏** |
|  | □ 3=了解 □ 2=了解一些 □ 1=不了解 |
|  | □ 3=非常重要 □ 2=比较重要 □ 1=不重要 |
|  | □ 3=完全做到 □ 2=有时做到 □ 1=从未做到  未完全做到的原因：□增加工作量 □做与不做效果一样  □想到做就做 □其他:____________ |
| **21** | **太极拳可用于肿瘤患者癌因性疲乏的管理** |
|  | □ 3=了解 □ 2=了解一些 □ 1=不了解 |
|  | □ 3=非常重要 □ 2=比较重要 □ 1=不重要 |
|  | □ 3=完全做到 □ 2=有时做到 □ 1=从未做到  未完全做到的原因：□增加工作量 □做与不做效果一样  □想到做就做 □其他:____________ |
| **22** | **可采用音乐疗法（如传统的五音疗法）缓解肿瘤患者的疲乏** |
|  | □ 3=了解 □ 2=了解一些 □ 1=不了解 |
|  | □ 3=非常重要 □ 2=比较重要 □ 1=不重要 |
|  | □ 3=完全做到 □ 2=有时做到 □ 1=从未做到  未完全做到的原因：□增加工作量 □做与不做效果一样  □想到做就做 □其他:____________ |
| 对症处理 | |
| **23** | **在进行癌因性疲乏症状管理时，应及时对症处理因癌症本身或癌症治疗导致的白细胞降低、流感样症状** |
|  | □ 3=了解 □ 2=了解一些 □ 1=不了解 |
|  | □ 3=非常重要 □ 2=比较重要 □ 1=不重要 |
|  | □ 3=完全做到 □ 2=有时做到 □ 1=从未做到  未完全做到的原因：□增加工作量 □做与不做效果一样  □想到做就做 □其他:____________ |
| **24** | **在进行癌因性疲乏症状管理时，应及时对症处理因癌症本身或癌症治疗导致的严重的恶心呕吐、水/电解质平衡紊乱** |
|  | □ 3=了解 □ 2=了解一些 □ 1=不了解 |
|  | □ 3=非常重要 □ 2=比较重要 □ 1=不重要 |
|  | □ 3=完全做到 □ 2=有时做到 □ 1=从未做到  未完全做到的原因：□增加工作量 □做与不做效果一样  □想到做就做 □其他:____________ |
| **25** | **对伴发食欲减退或厌食的癌因性疲乏患者，应采用改善食欲的药物（如安宫黄体酮、醋酸甲地孕酮等），来缓解疲乏** |
|  | □ 3=了解 □ 2=了解一些 □ 1=不了解 |
|  | □ 3=非常重要 □ 2=比较重要 □ 1=不重要 |
|  | □ 3=完全做到 □ 2=有时做到 □ 1=从未做到  未完全做到的原因：□增加工作量 □做与不做效果一样  □想到做就做 □其他:____________ |
| **26** | **在进行癌因性疲乏症状管理时，应及时对症处理因癌症本身或癌症治疗导致的疼痛、并发症（如癌性胸水）** |
|  | □ 3=了解 □ 2=了解一些 □ 1=不了解 |
|  | □ 3=非常重要 □ 2=比较重要 □ 1=不重要 |
|  | □ 3=完全做到 □ 2=有时做到 □ 1=从未做到  未完全做到的原因：□增加工作量 □做与不做效果一样  □想到做就做 □其他:____________ |
| **27** | **对因贫血导致疲乏的肿瘤患者可采用造血生长因子（促红细胞生成素或阿法达贝汀）来进行治疗** |
|  | □ 3=了解 □ 2=了解一些 □ 1=不了解 |
|  | □ 3=非常重要 □ 2=比较重要 □ 1=不重要 |
|  | □ 3=完全做到 □ 2=有时做到 □ 1=从未做到  未完全做到的原因：□增加工作量 □做与不做效果一样  □想到做就做 □其他:____________ |
| **28** | **在上述由癌症本身或癌症治疗导致的症状或体征得到有效缓解的基础上，患者若仍主诉中度至重度的疲乏，应进一步评估分析其他方面的因素** |
|  | □ 3=了解 □ 2=了解一些 □ 1=不了解 |
|  | □ 3=非常重要 □ 2=比较重要 □ 1=不重要 |
|  | □ 3=完全做到 □ 2=有时做到 □ 1=从未做到  未完全做到的原因：□增加工作量 □做与不做效果一样  □想到做就做 □其他:____________ |
| **29** | **对伴有抑郁的癌因性疲乏患者，应及时进行抗抑郁治疗，在此基础上若疲乏未能有效缓解，应进一步分析是否存在其他影响因素，以有效管理患者的疲乏** |
|  | □ 3=了解 □ 2=了解一些 □ 1=不了解 |
|  | □ 3=非常重要 □ 2=比较重要 □ 1=不重要 |
|  | □ 3=完全做到 □ 2=有时做到 □ 1=从未做到  未完全做到的原因：□增加工作量 □做与不做效果一样  □想到做就做 □其他:____________ |
| **30** | **对某些因行为因素和/或心理社会因素（如过分担心疾病复发、采用不恰当的应对方式等）导致癌因性疲乏的患者，可采用认知行为疗法、团体支持-表达治疗、正念减压法对其进行癌因性疲乏的管理** |
|  | □ 3=了解 □ 2=了解一些 □ 1=不了解 |
|  | □ 3=非常重要 □ 2=比较重要 □ 1=不重要 |
|  | □ 3=完全做到 □ 2=有时做到 □ 1=从未做到  未完全做到的原因：□增加工作量 □做与不做效果一样  □想到做就做 □其他:____________ |
| **31** | **对伴有睡眠障碍的癌因性疲乏患者可采用行为认知疗法、必要时遵医嘱结合安眠药物进行干预** |
|  | □ 3=了解 □ 2=了解一些 □ 1=不了解 |
|  | □ 3=非常重要 □ 2=比较重要 □ 1=不重要 |
|  | □ 3=完全做到 □ 2=有时做到 □ 1=从未做到  未完全做到的原因：□增加工作量 □做与不做效果一样  □想到做就做 □其他:____________ |
| **32** | **哌醋甲酯可考虑用于癌因性疲乏的治疗，但需长期服用才有效** |
|  | □ 3=了解 □ 2=了解一些 □ 1=不了解 |
|  | □ 3=非常重要 □ 2=比较重要 □ 1=不重要 |
|  | □ 3=完全做到 □ 2=有时做到 □ 1=从未做到  未完全做到的原因：□增加工作量 □做与不做效果一样  □想到做就做 □其他:____________ |
| **33** | **对重度疲乏患者可使用莫达非尼进行治疗** |
|  | □ 3=了解 □ 2=了解一些 □ 1=不了解 |
|  | □ 3=非常重要 □ 2=比较重要 □ 1=不重要 |
|  | □ 3=完全做到 □ 2=有时做到 □ 1=从未做到  未完全做到的原因：□增加工作量 □做与不做效果一样  □想到做就做 □其他:____________ |
| **支持治疗** | |
| **34** | **应该对癌因性疲乏患者进行营养风险筛查，并针对造成营养不良的原因采取相应的措施（如食欲减退者可采用增进食欲的药物）** |
|  | □ 3=了解 □ 2=了解一些 □ 1=不了解 |
|  | □ 3=非常重要 □ 2=比较重要 □ 1=不重要 |
|  | □ 3=完全做到 □ 2=有时做到 □ 1=从未做到  未完全做到的原因：□增加工作量 □做与不做效果一样  □想到做就做 □其他:____________ |
| **35** | **可遵医嘱使用益气扶正的中药对癌因性疲乏患者进行支持治疗** |
|  | □ 3=了解 □ 2=了解一些 □ 1=不了解 |
|  | □ 3=非常重要 □ 2=比较重要 □ 1=不重要 |
|  | □ 3=完全做到 □ 2=有时做到 □ 1=从未做到  未完全做到的原因：□增加工作量 □做与不做效果一样  □想到做就做 □其他:____________ |
