# Supplementary material for: Implementation of evidence into practice for cancer-related fatigue management of hospitalized adult patients using the PARIHS framework
Source: PLoS One. 2017 Oct 31;12(10):e0187257. doi: 10.1371/journal.pone.0187257 (PMC5663504; doi:10.1371/journal.pone.0187257)
Supplement: S4 Table — (DOCX) [file pone.0187257.s004.docx]

**Nurses’ knowledge, attitudes and behaviors regarding CRF nursing**

Thanks for your participation in this survey, which focus on your knowledge, attitudes and behaviors regarding CRF nursing, please tick (“√”)the box before the option most suitable to you.

1. Date of birth:________(month)/________(year)

2. When you came to work in the current ward: ________(month)/________(year)

3.Your current education background:

□postgraduate □university □junior college □technical secondary school

4. Your current professional titles：

□chief superintendent nurse □co-chief superintendent nurse □charge nurse □senior nurse □nurse

5.Your current position：□head nurse □nurse preceptor □nurse

| **No.** | **Item** |
| --- | --- |
| Screening and assessment | |
| **1** | **The nurse should screen CRF of the patients during their hospital visit.** |
|  | □3= fully aware □2= partly aware □ 1= unaware |
|  | □ 3=very important □ 2=relatively important □ 1=unimportant |
|  | □ 3=do completely □ 2=sometimes do □ 1=never do  Reasons for “not do completely”：□increase workload □there’s no difference between doing and not doing □do it when it just comes to mind □others:____________ |
| **2** | **CRF could be assessed by the patients themselves.** |
|  | □3= fully aware □2= partly aware □ 1= unaware |
|  | □ 3=very important □ 2=relatively important □ 1=unimportant |
|  | □ 3=do completely □ 2=sometimes do □ 1=never do  Reasons for “not do completely”：□increase workload □there’s no difference between doing and not doing □do it when it just come into the mind □other:____________ |
| **3** | **The nurse could teach the patient the methods of self-assessed CRF.** |
|  | □3= fully aware □2= partly aware □ 1= unaware |
|  | □ 3=very important □ 2=relatively important □ 1=unimportant |
|  | □ 3=do completely □ 2=sometimes do □ 1=never do  Reasons for “not do completely”：□increase workload □there’s no difference between doing and not doing □do it when it just come into the mind □other:____________ |
| **4** | **ICD-10-CRF criteria should be used to screen CRF.** |
|  | □3= fully aware □2= partly aware □ 1= unaware |
|  | □ 3=very important □ 2=relatively important □ 1=unimportant |
|  | □ 3=do completely □ 2=sometimes do □ 1=never do  Reasons for “not do completely”：□increase workload □there’s no difference between doing and not doing □do it when it just come into the mind □other:____________ |
| **5** | **Medical professionals should screen and assess CRF daily during the anti-cancer treatment.** |
|  | □3= fully aware □2= partly aware □ 1= unaware |
|  | □ 3=very important □ 2=relatively important □ 1=unimportant |
|  | □ 3=do completely □ 2=sometimes do □ 1=never do  Reasons for “not do completely”：□increase workload □there’s no difference between doing and not doing □do it when it just come into the mind □other:____________ |
| **6** | **Medical professionals should assess the patient’s CRF at regular intervals after the anti-cancer treatment ends.** |
|  | □3= fully aware □2= partly aware □ 1= unaware |
|  | □ 3=very important □ 2=relatively important □ 1=unimportant |
|  | □ 3=do completely □ 2=sometimes do □ 1=never do  Reasons for “not do completely”：□increase workload □there’s no difference between doing and not doing □do it when it just come into the mind □other:____________ |
| **7** | **0-10 numeric rating scale could be used when CRF needs to be assessed repeatedly and frequently. Can or could?** |
|  | □3= fully aware □2= partly aware □ 1= unaware |
|  | □ 3=very important □ 2=relatively important □ 1=unimportant |
|  | □ 3=do completely □ 2=sometimes do □ 1=never do  Reasons for “not do completely”：□increase workload □there’s no difference between doing and not doing □do it when it just come into the mind □other:____________ |
| **8** | **When doing comprehensive assessment and evaluating the effect of interventions, we can select appropriate assessment tools according to characteristics of the target population and frequency of the assessment.** |
|  | □3= fully aware □2= partly aware □ 1= unaware |
|  | □ 3=very important □ 2=relatively important □ 1=unimportant |
|  | □ 3=do completely □ 2=sometimes do □ 1=never do  Reasons for “not do completely”：□increase workload □there’s no difference between doing and not doing □do it when it just come into the mind □other:____________ |
| **9** | **Comprehensive evaluation of the influencing factors should be carried out to those patients with moderate or severe CRF, to identify the inducing or aggravating factors of CRF, and adopt corresponding interventions timely.** |
|  | □3= fully aware □2= partly aware □ 1= unaware |
|  | □ 3=very important □ 2=relatively important □ 1=unimportant |
|  | □ 3=do completely □ 2=sometimes do □ 1=never do  Reasons for “not do completely”：□increase workload □there’s no difference between doing and not doing □do it when it just come into the mind □other:____________ |
| **10** | **The risk factors of CRF reported by the patient should be assessed and confirmed.** |
|  | □3= fully aware □2= partly aware □ 1= unaware |
|  | □ 3=very important □ 2=relatively important □ 1=unimportant |
|  | □ 3=do completely □ 2=sometimes do □ 1=never do  Reasons for “not do completely”：□increase workload □there’s no difference between doing and not doing □do it when it just come into the mind □other:____________ |
| Intervention | |
| General interventions | |
| **11** | **Health education and counseling focused on CRF-related knowledge should be given to the patient, including assessment methods, influencing factors and interventions, etc.** |
|  | □3= fully aware □2= partly aware □ 1= unaware |
|  | □ 3=very important □ 2=relatively important □ 1=unimportant |
|  | □ 3=do completely □ 2=sometimes do □ 1=never do  Reasons for “not do completely”：□increase workload □there’s no difference between doing and not doing □do it when it just come into the mind □other:____________ |
| **12** | **Personalized health education should be used to manage the patient’s CRF.** |
|  | □3= fully aware □2= partly aware □ 1= unaware |
|  | □ 3=very important □ 2=relatively important □ 1=unimportant |
|  | □ 3=do completely □ 2=sometimes do □ 1=never do  Reasons for “not do completely”：□increase workload □there’s no difference between doing and not doing □do it when it just come into the mind □other:____________ |
| **13** | **Exercise therapy should be used to manage CRF of patients with non hematologic neoplasm, especially that aerobics could be used in breast cancer or prostate cancer patients.** |
|  | □3= fully aware □2= partly aware □ 1= unaware |
|  | □ 3=very important □ 2=relatively important □ 1=unimportant |
|  | □ 3=do completely □ 2=sometimes do □ 1=never do  Reasons for “not do completely”：□increase workload □there’s no difference between doing and not doing □do it when it just come into the mind □other:____________ |
| **14** | **Exercise compliance is crucial to the effect of exercise therapy.** |
|  | □3= fully aware □2= partly aware □ 1= unaware |
|  | □ 3=very important □ 2=relatively important □ 1=unimportant |
|  | □ 3=do completely □ 2=sometimes do □ 1=never do  Reasons for “not do completely”：□increase workload □there’s no difference between doing and not doing □do it when it just come into the mind □other:____________ |
| **15** | **Providing more support (e.g. professional guidance or counseling by medical professionals) to the patients undergoing anti-cancer treatments could improve their exercise compliance.** |
|  | □3= fully aware □2= partly aware □ 1= unaware |
|  | □ 3=very important □ 2=relatively important □ 1=unimportant |
|  | □ 3=do completely □ 2=sometimes do □ 1=never do  Reasons for “not do completely”：□increase workload □there’s no difference between doing and not doing □do it when it just come into the mind □other:____________ |
| **16** | **Providing relevant printed materials and exercise level monitoring instruments (e.g. pedometer) to patients receiving exercise therapy can improve their exercise level.** |
|  | □3= fully aware □2= partly aware □ 1= unaware |
|  | □ 3=very important □ 2=relatively important □ 1=unimportant |
|  | □ 3=do completely □ 2=sometimes do □ 1=never do  Reasons for “not do completely”：□increase workload □there’s no difference between doing and not doing □do it when it just come into the mind □other:____________ |
| **17** | **Patients with the following conditions should use the exercise therapy with caution: bone metastasis, thrombocytopenia, anemia, fever, acute infection, or limitations of movement secondary to metastasis.** |
|  | □3= fully aware □2= partly aware □ 1= unaware |
|  | □ 3=very important □ 2=relatively important □ 1=unimportant |
|  | □ 3=do completely □ 2=sometimes do □ 1=never do  Reasons for “not do completely”：□increase workload □there’s no difference between doing and not doing □do it when it just come into the mind □other:____________ |
| **18** | **Acupuncture and acupressure could be used to alleviate the patient’s CRF.** |
|  | □3= fully aware □2= partly aware □ 1= unaware |
|  | □ 3=very important □ 2=relatively important □ 1=unimportant |
|  | □ 3=do completely □ 2=sometimes do □ 1=never do  Reasons for “not do completely”：□increase workload □there’s no difference between doing and not doing □do it when it just come into the mind □other:____________ |
| **19** | **Local moxibustion could be used to alleviate the patient’s CRF.** |
|  | □3= fully aware □2= partly aware □ 1= unaware |
|  | □ 3=very important □ 2=relatively important □ 1=unimportant |
|  | □ 3=do completely □ 2=sometimes do □ 1=never do  Reasons for “not do completely”：□increase workload □there’s no difference between doing and not doing □do it when it just come into the mind □other:____________ |
| **20** | **Myofascial massage could be used to alleviate CRF of the reconvalescent.** |
|  | □3= fully aware □2= partly aware □ 1= unaware |
|  | □ 3=very important □ 2=relatively important □ 1=unimportant |
|  | □ 3=do completely □ 2=sometimes do □ 1=never do  Reasons for “not do completely”：□increase workload □there’s no difference between doing and not doing □do it when it just come into the mind □other:____________ |
| **21** | **Shadowboxing could be used to manage the patient’s CRF.** |
|  | □3= fully aware □2= partly aware □ 1= unaware |
|  | □ 3=very important □ 2=relatively important □ 1=unimportant |
|  | □ 3=do completely □ 2=sometimes do □ 1=never do  Reasons for “not do completely”：□increase workload □there’s no difference between doing and not doing □do it when it just come into the mind □other:____________ |
| **22** | **We can use music therapy (e.g. five-tone therapy in TCM) to alleviate the patient’s CRF** |
|  | □3= fully aware □2= partly aware □ 1= unaware |
|  | □ 3=very important □ 2=relatively important □ 1=unimportant |
|  | □ 3=do completely □ 2=sometimes do □ 1=never do  Reasons for “not do completely”：□increase workload □there’s no difference between doing and not doing □do it when it just come into the mind □other:____________ |
| Symptomatic treatment | |
| **23** | **During CRF management, we should carry out timely symptom management on low white blood cell count , influenza-like symptoms caused by cancer or its treatment.** |
|  | □3= fully aware □2= partly aware □ 1= unaware |
|  | □ 3=very important □ 2=relatively important □ 1=unimportant |
|  | □ 3=do completely □ 2=sometimes do □ 1=never do  Reasons for “not do completely”：□increase workload □there’s no difference between doing and not doing □do it when it just come into the mind □other:____________ |
| **24** | **During CRF management, we should carry out timely symptom management on severe nausea and vomiting, fluid and electrolyte imbalances caused by cancer or its treatment.** |
|  | □3= fully aware □2= partly aware □ 1= unaware |
|  | □ 3=very important □ 2=relatively important □ 1=unimportant |
|  | □ 3=do completely □ 2=sometimes do □ 1=never do  Reasons for “not do completely”：□increase workload □there’s no difference between doing and not doing □do it when it just come into the mind □other:____________ |
| **25** | **Appetite stimulants (e.g. medroxyprogesterone acetate, megestrol acetate, etc.) should be used to relieve CRF of patients with anorexia or apositia.** |
|  | □3= fully aware □2= partly aware □ 1= unaware |
|  | □ 3=very important □ 2=relatively important □ 1=unimportant |
|  | □ 3=do completely □ 2=sometimes do □ 1=never do  Reasons for “not do completely”：□increase workload □there’s no difference between doing and not doing □do it when it just come into the mind □other:____________ |
| **26** | **During CRF management, we should carry out timely symptom management on pain and complications (e.g. malignant pleural effusion) caused by cancer or its treatment.** |
|  | □3= fully aware □2= partly aware □ 1= unaware |
|  | □ 3=very important □ 2=relatively important □ 1=unimportant |
|  | □ 3=do completely □ 2=sometimes do □ 1=never do  Reasons for “not do completely”：□increase workload □there’s no difference between doing and not doing □do it when it just come into the mind □other:____________ |
| **27** | **Hemopoietic growth factor (e.g. erythropoietin or darbepoetin alpha) could be used to treat the patient’s CRF caused by anemia.** |
|  | □3= fully aware □2= partly aware □ 1= unaware |
|  | □ 3=very important □ 2=relatively important □ 1=unimportant |
|  | □ 3=do completely □ 2=sometimes do □ 1=never do  Reasons for “not do completely”：□increase workload □there’s no difference between doing and not doing □do it when it just come into the mind □other:____________ |
| **28** | **When symptoms or signs caused by cancer or its treatment are effectively managed, the patient still report moderate to severe CRF, then other relevant factors should be assessed and analyzed.** |
|  | □3= fully aware □2= partly aware □ 1= unaware |
|  | □ 3=very important □ 2=relatively important □ 1=unimportant |
|  | □ 3=do completely □ 2=sometimes do □ 1=never do  Reasons for “not do completely”：□increase workload □there’s no difference between doing and not doing □do it when it just come into the mind □other:____________ |
| **29** | **Anti-depression treatment should be adopted in CRF patients with depression. Based on this, if CRF do not relieve, other contributing factors of CRF should be analyzed to ensure effective CRF management.** |
|  | □3= fully aware □2= partly aware □ 1= unaware |
|  | □ 3=very important □ 2=relatively important □ 1=unimportant |
|  | □ 3=do completely □ 2=sometimes do □ 1=never do  Reasons for “not do completely”：□increase workload □there’s no difference between doing and not doing □do it when it just come into the mind □other:____________ |
| **30** | **Cognitive and behavioral therapy, group supportive-expressive therapy and mindfulness-based stress reduction could be used to manage the patient’s CRF induced by behavioral or psychosocial factors, such as excessive worries about recurrence, inappropriate coping mechanisms.** |
|  | □3= fully aware □2= partly aware □ 1= unaware |
|  | □ 3=very important □ 2=relatively important □ 1=unimportant |
|  | □ 3=do completely □ 2=sometimes do □ 1=never do  Reasons for “not do completely”：□increase workload □there’s no difference between doing and not doing □do it when it just come into the mind □other:____________ |
| **31** | **Cognitive and behavioral therapy could be used to intervene the CRF patient with sleep disorder, if necessary combined with hypnotics following the doctor’s advice** |
|  | □3= fully aware □2= partly aware □ 1= unaware |
|  | □ 3=very important □ 2=relatively important □ 1=unimportant |
|  | □ 3=do completely □ 2=sometimes do □ 1=never do  Reasons for “not do completely”：□increase workload □there’s no difference between doing and not doing □do it when it just come into the mind □other:____________ |
| **32** | **Methylphenidate could be used to treat CRF, but it works only with long-term use.** |
|  | □3= fully aware □2= partly aware □ 1= unaware |
|  | □ 3=very important □ 2=relatively important □ 1=unimportant |
|  | □ 3=do completely □ 2=sometimes do □ 1=never do  Reasons for “not do completely”：□increase workload □there’s no difference between doing and not doing □do it when it just come into the mind □other:____________ |
| **33** | **Modafinil could be used to treat the patient with severe CRF.** |
|  | □3= fully aware □2= partly aware □ 1= unaware |
|  | □ 3=very important □ 2=relatively important □ 1=unimportant |
|  | □ 3=do completely □ 2=sometimes do □ 1=never do  Reasons for “not do completely”：□increase workload □there’s no difference between doing and not doing □do it when it just come into the mind □other:____________ |
| **Supportive treatment** | |
| **34** | **Nutrition risk screening should be carried out in CRF patients, and the corresponding interventions on the causes of malnutrition should be adopted, e.g. giving appetite stimulants to patients with anorexia.** |
|  | □3= fully aware □2= partly aware □ 1= unaware |
|  | □ 3=very important □ 2=relatively important □ 1=unimportant |
|  | □ 3=do completely □ 2=sometimes do □ 1=never do  Reasons for “not do completely”：□increase workload □there’s no difference between doing and not doing □do it when it just come into the mind □other:____________ |
| **35** | **Chinese herbs that benefit and strengthen *qi* could be used following the doctor’s advice as the supportive treatment of CRF.** |
|  | □3= fully aware □2= partly aware □ 1= unaware |
|  | □ 3=very important □ 2=relatively important □ 1=unimportant |
|  | □ 3=do completely □ 2=sometimes do □ 1=never do  Reasons for “not do completely”：□increase workload □there’s no difference between doing and not doing □do it when it just come into the mind □other:____________ |
