# Supplementary material for: Implementation of evidence into practice for cancer-related fatigue management of hospitalized adult patients using the PARIHS framework
Source: PLoS One. 2017 Oct 31;12(10):e0187257. doi: 10.1371/journal.pone.0187257 (PMC5663504; doi:10.1371/journal.pone.0187257)
Supplement: S5 Table — (DOCX) [file pone.0187257.s005.docx]

**患者疲乏自我管理（知识、关注度、行为）调查表**

| 序 号 | 条 目 |
| --- | --- |
| **1** | **疾病本身或疾病的治疗会导致疲乏，对此我______________** |
|  | □ 3=了解 □ 2=了解一些 □ 1=不了解 |
|  | □ 3=非常关注 □ 2=比较关注 □ 1=不关注 |
|  | □ 3=经常会考虑 □ 2=有时会考虑 □ 1=从未考虑过 |
| **2** | **疲乏可由患者自己进行评估, 对此我______________** |
|  | □ 3=了解 □ 2=了解一些 □ 1=不了解 |
|  | □ 3=非常关注 □ 2=比较关注 □ 1=不关注 |
|  | □ 3=完全做到 □ 2=有时做到 □ 1=从未做到  未完全做到的原因： □疲乏不是我关心的重点 □汇报与不汇报效果一样  □医护人员不关心我的疲乏 □我不知该如何评估疲乏 □其他:_____________________ |
| **3** | **对放、化疗等导致的疲乏程度加重和疾病严重程度之间的关系，我____________________** |
|  | □ 3=了解 □ 2=了解一些 □ 1=不了解 |
|  | □ 3=非常关注 □ 2=比较关注 □ 1=不关注 |
|  | □ 3=经常会考虑 □ 2=有时会考虑 □ 1=从未考虑过 |
| **4** | **对引起或加重疲乏的因素，我______________** |
|  | □ 3=了解 □ 2=了解一些 □ 1=不了解 |
|  | □ 3=非常关注 □ 2=比较关注 □ 1=不关注 |
|  | □ 3=能完全应对 □ 2=能部分应对 □ 1=不知如何应对  未能完全应对的原因：□没有相关信息来源 □应对与不应对效果一样  □想到做就做 □其他:__________________________ |
| **5** | **在疾病治疗期间，需每天向医护人员报告我的疲乏程度, 对此我______________** |
|  | □ 3=了解 □ 2=了解一些 □ 1=不了解 |
|  | □ 3=非常关注 □ 2=比较关注 □ 1=不关注 |
|  | □ 3=完全做到 □ 2=有时做到 □ 1=从未做到  未完全做到的原因：□疲乏不是我关心的重点 □汇报与不汇报效果一样  □医护人员不关心我的疲乏 □我不知该如何汇报疲乏 □其他:____________________ |
| **6** | **疾病治疗结束，休养期间需继续评估记录疲乏的程度, 对此我______________** |
|  | □ 3=了解 □ 2=了解一些 □ 1=不了解 |
|  | □ 3=非常关注 □ 2=比较关注 □ 1=不关注 |
|  | □ 3=完全做到 □ 2=有时做到 □ 1=从未做到  未完全做到的原因：□疲乏不是我关心的重点 □关注与不关注效果一样  □医护人员不关心我的疲乏 □我不知该如何评估记录疲乏 □其他:_________________ |
| **7** | **疾病或疾病治疗引起的白细胞降低、贫血、严重的恶心呕吐、食欲不好、胸水等会加重疲**  **乏，需要报告医护人员及时对症处理, 对此我______________** |
|  | □ 3=了解 □ 2=了解一些 □ 1=不了解 |
|  | □ 3=非常关注 □ 2=比较关注 □ 1=不关注 |
|  | □ 3=完全做到 □ 2=有时做到 □ 1=从未做到  未完全做到的原因：□疲乏不是我关心的重点 □医护人员不关心我的疲乏  □这些都是正常反应，关注与不关注效果一样 □其他:_________________ |
| **8** | **运动疗法（如步行、跳舞、骑车、太极拳等）可以缓解疲乏, 对此我______________** |
|  | □ 3=了解 □ 2=了解一些 □ 1=不了解 |
|  | □ 3=非常关注 □ 2=比较关注 □ 1=不关注 |
|  | □ 3=经常运动 □ 2=有时运动 □ 1=从不运动  不运动的原因：□我不喜欢运动 □运动与不运动效果一样 □运动会加重疲乏  □我不知该如何做运动 □其他:____________________ |
| **9** | **有些情况（如骨转移、血小板减少症、贫血、发热或急性感染等）是不宜做运动的, 对此我______________** |
|  | □ 3=了解 □ 2=了解一些 □ 1=不了解 |
|  | □ 3=非常关注 □ 2=比较关注 □ 1=不关注 |
|  | □ 3=经常想到 □ 2=有时想到 □ 1=从未想到  想不到的原因：□没有相关信息来源 □我觉得出现症状后运动没什么关系  □其他:________________________________ |
| **10** | **物理疗法（如针灸、艾灸、按摩等）可以缓解疲乏, 对此我______________** |
|  | □ 3=了解 □ 2=了解一些 □ 1=不了解 |
|  | □ 3=非常关注 □ 2=比较关注 □ 1=不关注 |
|  | □ 4=经常采用 □ 3=有时采用 □ 1=从未采用  未采用该疗法的原因：□没有相关信息来源 □不知道去哪里获得这些治疗  □这些方法用与不用效果一样 □需要经常去医院，不方便 □其他:______________ |
| **11** | **音乐疗法（如唱歌、听音乐等）可以缓解疲乏, 对此我______________** |
|  | □ 3=了解 □ 2=了解一些 □ 1=不了解 |
|  | □ 3=非常关注 □ 2=比较关注 □ 1=不关注 |
|  | □ 3=经常采用 □ 2=有时采用 □ 1=从未采用  未采用该疗法的原因：□没有相关信息来源 □不喜欢音乐  □用与不用效果一样 □其他:______________ |
| **12** | **管理好情绪（如焦虑、抑郁）有助于缓解疲乏, 对此我______________** |
|  | □ 3=了解 □ 2=了解一些 □ 1=不了解 |
|  | □ 3=非常关注 □ 2=比较关注 □ 1=不关注 |
|  | □ 3=完全做到 □ 2=有时做到 □ 1=从未做到  未完全做到的原因：□天生性格就这样，管理与不管理效果一样  □我不知该如何管理自己的情绪 □其他:____________________ |
| **13** | **寻求专业团队的支持和帮助有助于管理疲乏, 对此我______________** |
|  | □ 3=了解 □ 2=了解一些 □ 1=不了解 |
|  | □ 3=非常关注 □ 2=比较关注 □ 1=不关注 |
|  | □ 3=经常采用 □ 2=有时采用 □ 1=从未采用  未采用该方法的原因：□没有相关社会资源 □用与不用效果一样  □其他:____________________ |
| **14** | **寻求家人、朋友的支持和帮助有助于管理疲乏, 对此我______________** |
|  | □ 3=了解 □ 2=了解一些 □ 1=不了解 |
|  | □ 3=非常关注 □ 2=比较关注 □ 1=不关注 |
|  | □ 3=经常采用 □ 2=有时采用 □ 1=从未采用  未采用该方法的原因：□家人、朋友都很忙，没时间听我倾诉  □用与不用效果一样 □其他:____________________ |
| **15** | **保证良好的睡眠有助于缓解我的疲乏, 对此我______________** |
|  | □ 3=了解 □ 2=了解一些 □ 1=不了解 |
|  | □ 3=非常关注 □ 2=比较关注 □ 1=不关注 |
|  | □ 3=完全做到 □ 2=有时做到 □ 1=从未做到  未完全做到的原因：□没有相关信息来源 □睡眠质量变差是正常现象，不需要处理  □没有有效的办法能提高睡眠质量 □其他:_______________ |
| **16** | **服用益气扶正的中药有助于缓解疲乏, 对此我______________** |
|  | □ 3=了解 □ 2=了解一些 □ 1=不了解 |
|  | □ 3=非常关注 □ 2=比较关注 □ 1=不关注 |
|  | □ 3=经常采用 □ 2=有时采用 □ 1=从未采用  未采用该疗法的原因：□受不了中药的味道、吃不下去 □不知去哪里配这样的中药  □用与不用效果一样 □其他:____________________ |
