# Supplementary material for: Implementation of evidence into practice for cancer-related fatigue management of hospitalized adult patients using the PARIHS framework
Source: PLoS One. 2017 Oct 31;12(10):e0187257. doi: 10.1371/journal.pone.0187257 (PMC5663504; doi:10.1371/journal.pone.0187257)
Supplement: S6 Table — (DOCX) [file pone.0187257.s006.docx]

**Patients’ self-management (knowledge, attitude and behaviors) scale**

| No. | Item |
| --- | --- |
| **1** | **Cancer or its treatments can result in CRF, for this I**______ |
|  | □ 3= aware □ 2=partly aware □ 1=unknown unaware |
|  | □ 3=very concerned □ 2=relatively concerned □ 1=not concerned |
|  | □ 3=often consider □ 2=sometimes consider □ 1=never consider |
| **2** | **CRF can be self-assessed by patients, for this I**______ |
|  | □ 3= aware □ 2=partly aware □ 1=unknown unaware |
|  | □ 3=very concerned □ 2=relatively concerned □ 1=not concerned |
|  | □ 3=do completely □ 2=sometimes do □ 1=never do  Reasons for “not do completely”： □CRF is not the point I care about □ there’s no difference between reporting and not reporting □medical professionals do not care my CRF □I don’t know how to assess my CRF □other:_______________ |
| **3** | **For the correlation between the aggravating CRF induced by radiotherapy or chemotherapy and severity of illness, I**______ |
|  | □ 3= aware □ 2=partly aware □ 1=unknown unaware |
|  | □ 3=very concerned □ 2=relatively concerned □ 1=not concerned |
|  | □ 3=often consider □ 2=sometimes consider □ 1=never consider |
| **4** | **For those CRF inducing or aggravating factors, I**______ |
|  | □ 3= aware □ 2=partly aware □ 1=unknown unaware |
|  | □ 3=very concerned □ 2=relatively concerned □ 1=not concerned |
|  | □ 3= cope completely □ 2=cope partly □ 1=do not know how to cope  Reasons for “not cope completely”：□ having no relevant source of information □ there’s no difference between coping and not coping □ cope with it when it just comes to mind □ others:______________ |
| **5** | **I need to report my CRF daily to the medical professionals during the anti-cancer treatment, for this I**______ |
|  | □ 3= aware □ 2=partly aware □ 1=unknown unaware |
|  | □ 3=very concerned □ 2=relatively concerned □ 1=not concerned |
|  | □ 3=do completely □ 2=sometimes do □ 1=never do  Reasons for “not do completely”： □CRF is not the focus of my attention □ there’s no difference between reporting and not reporting □the medical professionals do not care about my CRF □I don’t know how to assess my CRF □other:_______________ |
| **6** | **CRF needs to be assessed and recorded after the anti-cancer treatment is completed, for this I**______ |
|  | □ 3= aware □ 2=partly aware □ 1=unknown unaware |
|  | □ 3=very concerned □ 2=relatively concerned □ 1=not concerned |
|  | □ 3=do completely □ 2=sometimes do □ 1=never do  Reasons for “not do completely”： □CRF is not the focus of my attention □ there’s no difference between reporting and not reporting □the medical professionals do not care about my CRF □I don’t know how to assess my CRF □other:_______________ |
| **7** | **Symptoms (e.g. low WBC count, anemia, severe nausea and vomiting, anorexia, pleural effusion, etc.) caused by cancer or its treatments can aggravate CRF, and need to be reported to the medical professionals so that they can adopt timely interventions, for this I**______ |
|  | □ 3= aware □ 2=partly aware □ 1=unknown unaware |
|  | □ 3=very concerned □ 2=relatively concerned □ 1=not concerned |
|  | □ 3=do completely □ 2=sometimes do □ 1=never do  Reasons for “not do completely”： □CRF is not the point I care about □medical professionals do not care my CRF □it’s normal reaction, there’s no difference between reporting and not reporting □other:_______________ |
| **8** | **Exercise therapy (e.g. walking, dancing, bicycling, shadowboxing, etc.) can alleviate CRF, for this I**______ |
|  | □ 3= aware □ 2=partly aware □ 1=unknown unaware |
|  | □ 3=very concerned □ 2=relatively concerned □ 1=not concerned |
|  | □ 3=often exercise □ 2=sometimes exercise □ 1=never exercise  Reasons for “never exercise”：□I dislike exercise □there’s no difference between exercise and no exercise □exercise can aggravate CRF □I don’t know how to exercise □ others:________ |
| **9** | **Some conditions (e.g. bone metastasis, thrombocytopenia, anemia, fever or acute infection, etc.) are not suitable for exercise therapy, for this I**______ |
|  | □ 3= aware □ 2=partly aware □ 1=unknown unaware |
|  | □ 3=very concerned □ 2=relatively concerned □ 1=not concerned |
|  | □ 3=often consider □ 2=sometimes consider □ 1=never consider  Reasons for “never consider”：□ without relevant information sources □it doesn’t matter to take exercise after these situation emerge □ others:______________ |
| **10** | **Physical therapies (e.g. acupuncture, moxibustion, massage, etc.) can relieve CRF, for this I**______ |
|  | □ 3= aware □ 2=partly aware □ 1=unknown unaware |
|  | □ 3=very concerned □ 2=relatively concerned □ 1=not concerned |
|  | □ 3=often use □ 2=sometimes use □ 1=never use  Reasons for “never use”:□ having no relevant source of information □don’t know how to get these therapies □ there’s no difference between using and not using □it’s inconvenient because these therapies need going to hospital frequently □ others:______________ |
| **11** | **Music therapy (e.g. singing, listening to music) can relieve CRF, for this I**______ |
|  | □ 3= aware □ 2=partly aware □ 1=unknown unaware |
|  | □ 3=very concerned □ 2=relatively concerned □ 1=not concerned |
|  | □ 3=often use □ 2=sometimes use □ 1=never use  Reasons for “never use”:□ having no relevant source of information □dislike music □ there’s no difference between using and not using □others:______________ |
| **12** | **Managing emotions (e.g. anxiety, depression) effectively is beneficial to the alleviation of CRF, for this I**______ |
|  | □ 3= aware □ 2=partly aware □ 1=unknown unaware |
|  | □ 3=very concerned □ 2=relatively concerned □ 1=not concerned |
|  | □ 3=do completely □ 2=sometimes do □ 1=never do  Reasons for “not do completely”：□ it is my personality and cannot be changed □I don’t know how to manage my emotions □ others:______________ |
| **13** | **Seeking support and help from professional teams is beneficial to CRF management, for this I**______ |
|  | □ 3= aware □ 2=partly aware □ 1=unknown unaware |
|  | □ 3=very concerned □ 2=relatively concerned □ 1=not concerned |
|  | □ 3=often use □ 2=sometimes use □ 1=never use  Reasons for “never use”：□ without relevant social sources □ there is no difference between using and not using □others:______________ |
| **14** | **Seeking support and help from family members and friends is beneficial to CRF management, for this I**______ |
|  | □ 3= aware □ 2=partly aware □ 1=unknown unaware |
|  | □ 3=very concerned □ 2=relatively concerned □ 1=not concerned |
|  | □ 3=often use □ 2=sometimes use □ 1=never use  Reasons for “never use:□ family members and friends are too busy to listen to me □ there’s no difference between using and not using □others:______________ |
| **15** | **Good sleep contributes to alleviation of CRF, for this I**______ |
|  | □ 3= aware □ 2=partly aware □ 1=unknown unaware |
|  | □ 3=very concerned □ 2=relatively concerned □ 1=not concerned |
|  | □ 3=do completely □ 2=sometimes do □ 1=never do  Reasons for “not do completely”:□ having no relevant source of information □the deterioration of sleep quality is normal and it is unnecessary to do something about it □there are no effective methods to improve sleep quality □other:_______________ |
| **16** | **Taking Chinese herbs that benefit and strengthen *qi* contribute to alleviation of CRF, for this I**______ |
|  | □ 3= aware □ 2=partly aware □ 1=unknown unaware |
|  | □ 3=very concerned □ 2=relatively concerned □ 1=not concerned |
|  | □ 3=often use □ 2=sometimes use □ 1=never use  Reasons for “never use”:□ cannot tolerate the taste of Chinese herbs □do not know where to get the herbs □there’s no difference between using and not using □others:______________ |
