# Supplementary material for: Implementation of evidence into practice for cancer-related fatigue management of hospitalized adult patients using the PARIHS framework
Source: PLoS One. 2017 Oct 31;12(10):e0187257. doi: 10.1371/journal.pone.0187257 (PMC5663504; doi:10.1371/journal.pone.0187257)
Supplement: S7 Table — (DOCX) [file pone.0187257.s007.docx]

**疲乏管理自我效能问卷**

您好！首先非常感谢您参加此项调查，我们承诺对您所提供的资料严格保密，问卷上不用填写您的名字，只以编号形式统计资料。答案没有对错之分，您只要按照真实情况填写即可，再次向您表示衷心的感谢！

**请您准确填写下列信息：**

住院号：_______________ 出生年月：_______年______月

性别：□男 □女 疾病诊断：____________________________

目前治疗：

□化疗 方案：_______________ 第_______次化疗

□放疗 方案：_______________ 第_______次放疗

**填表指导语：**以下条目主要是关于您疲乏后所面临的挑战，下列评分主要表示您面对挑战时的自信心，请您在最符合您的分值上打“√”。

**1. 您是否有信心应对疲乏对您生理上、心理上和精神上造成的压力**

**无信心 非常有信心**

|  |  |  |  |  |  |
| --- | --- | --- | --- | --- | --- |

0 1 2 3 4 5 6

**2. 您面对自己因疾病或疾病治疗导致疲乏这一事实的态度是**

**难以接受 能够接受**

|  |  |  |  |  |  |
| --- | --- | --- | --- | --- | --- |

0 1 2 3 4 5 6

**3. 出现疲乏后，您能否尽可能地保持正常的生活方式**

**不能够 完全能够**

|  |  |  |  |  |  |
| --- | --- | --- | --- | --- | --- |

0 1 2 3 4 5 6

**4. 您是否能够控制由于对将来的不确定感所带来的焦虑情绪**

**不能够 完全能够**

|  |  |  |  |  |  |
| --- | --- | --- | --- | --- | --- |

0 1 2 3 4 5 6

**5. 您是否有信心应对疲乏对日常活动（如洗澡、整理个人内务等）造成的影响**

**无信心 非常有信心**

|  |  |  |  |  |  |
| --- | --- | --- | --- | --- | --- |

0 1 2 3 4 5 6

**6. 您是否有信心处理疲乏对您的家庭所带来的影响（如因为疲乏，影响您或家庭成员的正常工作）**

**无信心 非常有信心**

|  |  |  |  |  |  |
| --- | --- | --- | --- | --- | --- |

0 1 2 3 4 5 6
