# Supplementary material for: Implementation of evidence into practice for cancer-related fatigue management of hospitalized adult patients using the PARIHS framework
Source: PLoS One. 2017 Oct 31;12(10):e0187257. doi: 10.1371/journal.pone.0187257 (PMC5663504; doi:10.1371/journal.pone.0187257)
Supplement: S8 Table — (DOCX) [file pone.0187257.s008.docx]

**Self-efficacy questionnaire for CRF management**

Thanks for your participation in this survey. We promise that your information would be kept confidential, and we collate using serial numbers instead of names. There is no true or false regarding the answer, and you need to fill out the form according to the facts. Thanks for your cooperation!

**Please fill out the following blanks accurately :**

Admission number：_______________ Date of birth：____(month)/_____(year)

Gender ：□male □female diagnosis：____________________

Current treatment：

□chemotherapy scheme：_______________ the No. of chemotherapy: _______

□radiotherapy scheme：_______________ the No. of radiotherapy: _______

**Instructions**: The following items are about the challenges you may encounter after you have cancer-related fatigue. The scale demonstrates your confidence for these challenges, please rate the most appropriate number with “√”.

1. whether you have confidence in coping with the physical, psychological and mental stress induced by fatigue?

no confidence full confidence

|  |  |  |  |  |  |
| --- | --- | --- | --- | --- | --- |

0 1 2 3 4 5 6

2. what is your attitude toward the fact that the disease and its treatment resulted in the fatigue?

hard to accept capable to accept

|  |  |  |  |  |  |
| --- | --- | --- | --- | --- | --- |

0 1 2 3 4 5 6

3. whether you could maintain your normal life style as much as possible after the fatigue emerged?

completely not completely yes

|  |  |  |  |  |  |
| --- | --- | --- | --- | --- | --- |

0 1 2 3 4 5 6

4. whether you could control the anxiety induced by the uncertainty about the future?

completely not completely yes

|  |  |  |  |  |  |
| --- | --- | --- | --- | --- | --- |

0 1 2 3 4 5 6

5. whether you have confidence in coping with the effect of fatigue on daily activities, such as bathing, personal hygiene?

no confidence full confidence

|  |  |  |  |  |  |
| --- | --- | --- | --- | --- | --- |

0 1 2 3 4 5 6

6. whether you have confidence in coping with the effect of fatigue on your family, such as fatigue influencing routine work of you or your family members?

no confidence full confidence

|  |  |  |  |  |  |
| --- | --- | --- | --- | --- | --- |

0 1 2 3 4 5 6
